# Defining the molecular response to ischemia-reperfusion injury and remote ischemic preconditioning in human kidney transplantation

**DOI:** 10.1371/journal.pone.0311613

**Published:** 2024-10-29

**Authors:** Johan Nordström, Pau Badia-I-Mompel, Anna Witasp, Angelina Schwarz, Pieter Evenepoel, Matthias B. Moor, Lars Wennberg, Julio Saez-Rodriguez, Annika Wernerson, Hannes Olauson

**Affiliations:** 1 Department of Transplantation Surgery, Karolinska University Hospital, Stockholm, Sweden; 2 Division of Transplantation Surgery, Department of Clinical Science, Intervention and Technology, Karolinska Institutet, Stockholm, Sweden; 3 Institute for Computational Biomedicine, Bioquant, Faculty of Medicine, and Heidelberg University Hospital, Heidelberg University, Heidelberg, Germany; 4 Division of Renal Medicine, Department of Clinical Science, Intervention, and Technology, Karolinska Institutet, Stockholm, Sweden; 5 Department of Microbiology, Immunology and Transplantation, Nephrology and Renal Transplantation Research Group, KU Leuven, Belgium; 6 Department of Nephrology and Renal Transplantation, University Hospitals Leuven, KU Leuven, Belgium; 7 Division of Pathology, Department of Laboratory Medicine, Karolinska Institutet, Stockholm, Sweden; Khalifa University of Science and Technology, UNITED ARAB EMIRATES

## Abstract

**Background:**

Ischemia-reperfusion injury (IRI) inevitably occurs during kidney transplantation and extended ischemia is associated with delayed graft function and poor outcomes. Remote ischemic preconditioning (RIPC) is a simple, noninvasive procedure aimed at reducing IRI and improving graft function. Experimental studies have implicated the kynurenine pathway as a protective mechanism behind RIPC.

**Methods:**

First, paired biopsies from 11 living kidney donors were analyzed to characterize the acute transcriptomic response to IRI. Second, 16 living kidney donors were subjected to either RIPC (n = 9) or no pretreatment (n = 7) to evaluate the impact of RIPC on the transcriptomic response to IRI. Finally, the effect of RIPC on plasma metabolites was analyzed in 49 healthy subjects.

**Results:**

There was a robust immediate response to IRI in the renal transcriptomes of living-donor kidney transplantation, including activation of the mitogen-activated protein kinase (MAPK) and epidermal growth factor receptor (EGFR) pathways. Preconditioning with RIPC did not significantly alter the transcriptomic response to IRI or the concentration of plasma metabolites.

**Conclusions:**

The present data validate living-donor kidney transplantation as a suitable model for mechanistic studies of IRI in human kidneys. The failure of RIPC to alter transcriptomic responses or metabolites in the kynurenine pathway raises the question of the robustness of the standard procedure used to induce RIPC, and might explain the mixed results in clinical trials evaluating RIPC as a method to attenuate IRI.

## Introduction

Globally, approximately two million people are affected by end-stage renal disease (ESRD) and require dialysis or kidney transplantation for survival. Kidney transplantation is the best treatment option in most patients. Due to the nature of the transplantation process, ischemia and reperfusion injury (IRI) of the kidney graft is inevitable. Significant IRI following kidney transplantation often manifests as delayed graft function (DGF), which has been reported to occur in 20–40% of all transplanted kidneys [1]. DGF has been reported to impair renal function, patient survival, graft survival and quality-adjusted life years [2]. In addition, DGF increases the risk of allograft rejection, prolongs hospital stay and increases healthcare costs. However, despite their significant negative impact on important clinical patient outcomes, effective therapies to prevent IRI in kidney transplantation are currently lacking. Safe and effective means of preventing or reducing IRI are of significant clinical importance and would presumably improve both the short- and long-term outcomes of kidney transplantation.

The protective effects of remote ischemic preconditioning (RIPC) were first demonstrated in 1993 in a dog model of myocardial infarction [3] and were subsequently followed by studies showing protection of other organs. Briefly, short and controlled episodes of non-harmful ischemia in body parts remote from the organ of interest, for example, in an arm, can protect against subsequent episodes of IRI in the target organ such as the heart, brain, or kidney. Several clinical trials have examined whether RIPC can reduce the risk of acute kidney injury in open-heart surgery and kidney transplantation with mixed results [4–7]. In the largest kidney transplantation RIPC trial to date, the REPAIR study, 406 living donor-recipient pairs were randomized to either RIPC by four five-minute episodes of ischemia to one arm, or no preconditioning [6]. Although the REPAIR study did not meet its primary outcome, differences in glomerular filtration rate (GFR) as measured by iohexol clearance and creatinine estimated GFR were significantly higher at 12 months in the group receiving early RIPC.

Most studies on molecular responses to IRI and RIPC have been performed in animal models, and only a few studies have explored the mechanisms of RIPC in human samples. Living-donor kidney transplantation offers a unique opportunity to study the immediate cellular response in humans to IRI and the effects of interventions such as RIPC under controlled and standardized conditions. Since living kidney donors are accepted based on their excellent general health status and kidney function, there is little confounding influence of pre-existing kidney injury.

In the present study, we generated a molecular fingerprint of the immediate transcriptomic response of the kidney to IRI in paired pre- and post-ischemic kidney biopsies from living donors. Next, we investigated whether the transcriptomic response to IRI was modulated by preoperative intervention with RIPC, which could provide clues to the mechanism underlying its putative protective effects. Finally, we assessed whether kynurenic acid (KYNA), a purported key mediator of RIPC, is affected by the current standard protocols to induce RIPC.

## Materials and methods

### Subject selection, kidney biopsies and blood sampling

Since 2015, all living kidney donors and kidney transplant recipients at Karolinska University Hospital, Stockholm, Sweden, have been invited to participate in a continuous local renal biopsy project with collection and biobanking of surplus kidney biopsy material. This project and the biobank were approved by the regional ethics committee (2015/1115-31, 2016/2057-32 and 2017/2353-32) and adhered to the statutes of the Declaration of Helsinki. The study was not blinded to either the participants or the researchers. All healthy subjects who volunteered to participate in the study signed a written informed consent form. In the study, 11 living kidney donor-recipient pairs were selected for the IRI biopsy group, and 23 pairs were selected for the RIPC biopsy group. The recruitment period lasted from September 1, 2015, to January 1, 2019.

In living kidney donors, the GFR was measured using iohexol clearance. The estimated glomerular filtration rate (eGFR) was calculated in recipients at baseline and 12 months after transplantation using the CKD-EPI 2021 equation.

All biopsies were obtained from the renal cortex using a biopsy needle (Temno™ 16 G × 6 cm CareFusion, Illinois, USA). A portion of the biopsies was immediately placed in RNAlater, frozen, and stored at –80°C. The remainder of the biopsy specimens were preserved in 4% formaldehyde. Before analysis, biopsies from the IRI cohort were micro-dissected into a glomerular and a tubulointerstitial fractions. In the RIPC cohort, biopsies were not micro-dissected. A total of 54 renal biopsy samples were subjected to RNA sequencing.

#### IRI biopsy group

**Fig 1:**
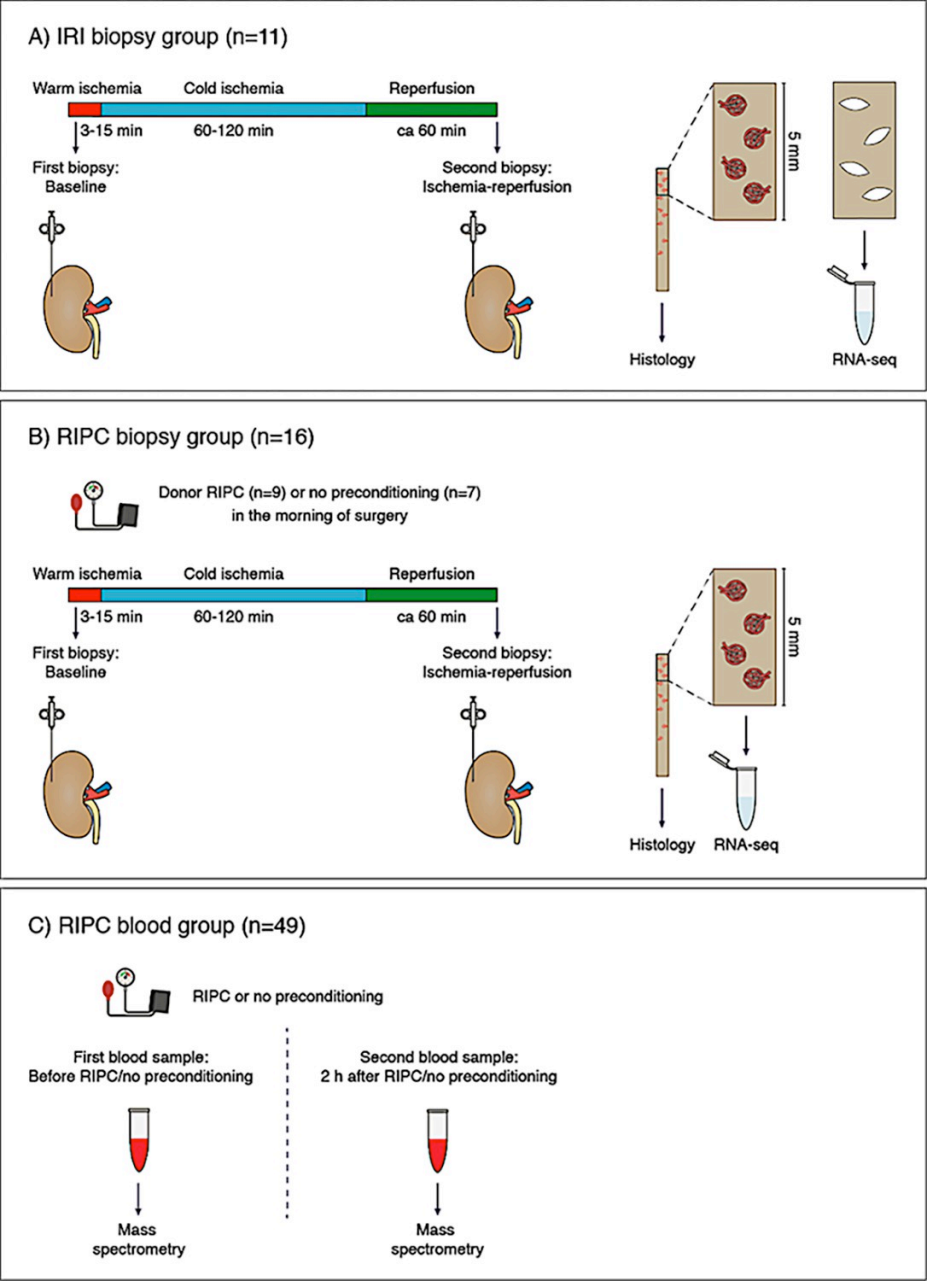
Study outlines. A) In the IRI biopsy group (n = 11 donor-recipient pairs) the kidney’s immediate cellular response to IRI was characterised by RNA sequencing in paired biopsies. During surgery, a first kidney biopsy was taken at back-table with 3–15 minutes of warm ischemia (Baseline) and a second biopsy was taken after 60–120 minutes of cold ischemia and approximately 60 minutes after reperfusion in the recipient (Ischemia-reperfusion). B) In the RIPC biopsy group (n = 16 donor-recipient pairs), nine donors received RIPC immediately prior to surgery and seven donors received no preconditioning, and the potential impact of RIPC on the response to IRI was evaluated by RNA sequencing. C) In the RIPC blood sample group, samples were collected from living donors (n = 23) at the morning of surgery and approximately 2 h after the first blood sample, and in healthy controls (n = 26) immediately before RIPC and 2 h later. Thereafter, the potential impact of RIPC on KYNA and other metabolites of the kynurenine pathway was analyzed using mass spectrometry.

Paired kidney biopsies were available from all 11 living kidney donor-recipient pairs. During surgery, the initial kidney biopsy, referred to as Baseline was obtained at the back-table under warm ischemic conditions lasting 3–15 minutes. Subsequently, a second biopsy, referred to as Ischemia-reperfusion, was performed approximately 60 min after reperfusion in the recipient, following ureteral anastomosis, at the end of the surgical procedure. The second biopsy was exposed to 60–120 min of cold ischemia. In total, two biopsies were obtained: one at the back-table (Baseline) and one following reperfusion (Ischemia-reperfusion) (Fig 1A).

#### RIPC biopsy group

Of the 23 pairs in the RIPC biopsy group, 16 paired kidney biopsies were available. In the remaining seven pairs, only one biopsy was available because the surgery continued after office hours; therefore, the second biopsy could not be analyzed and included. Of the donors in the RIPC biopsy group with paired biopsies available, nine donors were subjected to RIPC and seven donors did not receive any preconditioning. Biopsies were performed using the same methodology as that outlined for the IRI biopsy group (Fig 1B).

#### RIPC blood sample group

All living donors in the RIPC biopsy group were included in the RIPC blood sample cohort. Paired blood samples were obtained from all the 23 donors. Blood was sampled from living kidney donors on the morning of surgery, prior to RIPC, and approximately two h after the first blood sample (Fig 1C).

#### Healthy controls

In healthy controls (n = 26), blood samples were drawn as described for living donors (Fig 1C). Among the healthy controls, 13 were subjected to RIPC and 13 did not receive any preconditioning.

### RIPC procedure

The RIPC protocol consisted of four episodes of 5 minutes of ischemia in one arm induced by inflating a blood pressure cuff to 200 mmHg. In the control group, the subjects did not receive any preconditioning.

### Mass spectrometry

Blood samples from living donors and healthy subjects drawn before and after RIPC/no preconditioning were centrifuged and stored as serum at -80°C until analysis. Serum metabolites were measured using mass spectrometry as previously described [8]. Briefly, 50 μL serum sample was deproteinized with acetonitrile after the addition of stable isotope labelled internal standards and then filtered over a 96-well Ostro plate (Waters, Zellik, Belgium). After drying with nitrogen and redissolving in MilliQ water, the samples were chromatographically separated and detection was performed using a tandem mass spectrometer with alternating positive and negative electrospray ionization.

### Histology

Kidney biopsy material for light microscopy was immediately placed in 4% phosphate buffered formalin. After embedding in paraffin, 1–2 μm thick sections were cut and stained with hematoxylin and eosin (HE), Periodic Acid Schiff (PAS), and Ladewig stainings according to standard protocols. Biopsies were blindly evaluated by an experienced kidney pathologist (A.We). Interstitial inflammation, acute and chronic tubulointerstitial changes, and arteriolar hyalinosis were semi-quantified according to the Banff classification [9].

### Statistics

Data are expressed as the mean ± standard deviation (SD) or median with interquartile range (IQR) for continuous variables. A t-test or paired t-test was used to assess differences in clinical variables and metabolites from paired samples, respectively. Statistical analyses were performed using GraphPad Prism 8 for macOS. A p-value of <0.05 was considered statistically significant.

### RNA-sequencing analysis

RNA was extracted from the biopsies using RNeasy kits (Qiagen), and the quality was assessed using an Agilent 2100 Bioanalyzer (Agilent). An Illumina TruSeq Stranded mRNA sample preparation kit was used to prepare cDNA libraries from poly A-selected RNA samples. Libraries were sequenced on HiSeq2500. Basecalling and demultiplexing were performed using Illumina bcl2fastq (v1.8.4). Sequence data quality was assessed using FastQC (v0.11.8). The reads were aligned to the Ensembl GRCh38 reference genome using STAR (v2.6.1d). Counts for each gene were estimated using featureCounts (v1.5.1). The Bioconductor package DESeq2 (v1.34) was used for paired sample group comparisons, generating log2 fold changes, Wald test p-values and p-values adjusted for multiple testing (Benjamini-Hochberg method).

We leveraged the Multivariate Linear Model (MLM) method to infer transcription factor (TF) activities. Specifically, for each contrast identified through the previously performed DEA, we applied the MLM method implemented in the decoupleR/2.2.2 package [10]. The MLM method operates by fitting a linear model to predict observed gene expression changes based on the weights of TF-gene interactions extracted from the DoRoThEA gene regulatory network [11]. Subsequently, t-values derived from the slopes of the fitted models served as activity scores for the respective TFs. A positive score indicated TF activation, whereas a negative score indicated repression. Pathway activity inference was also performed by MLM, using the t-values obtained from DEA and the signature resource PROGENy filtered by the top 100 most significant responsive genes per pathway [12].

## Results

### Patient characteristics and histology in the IRI biopsy group

**Fig 2:**
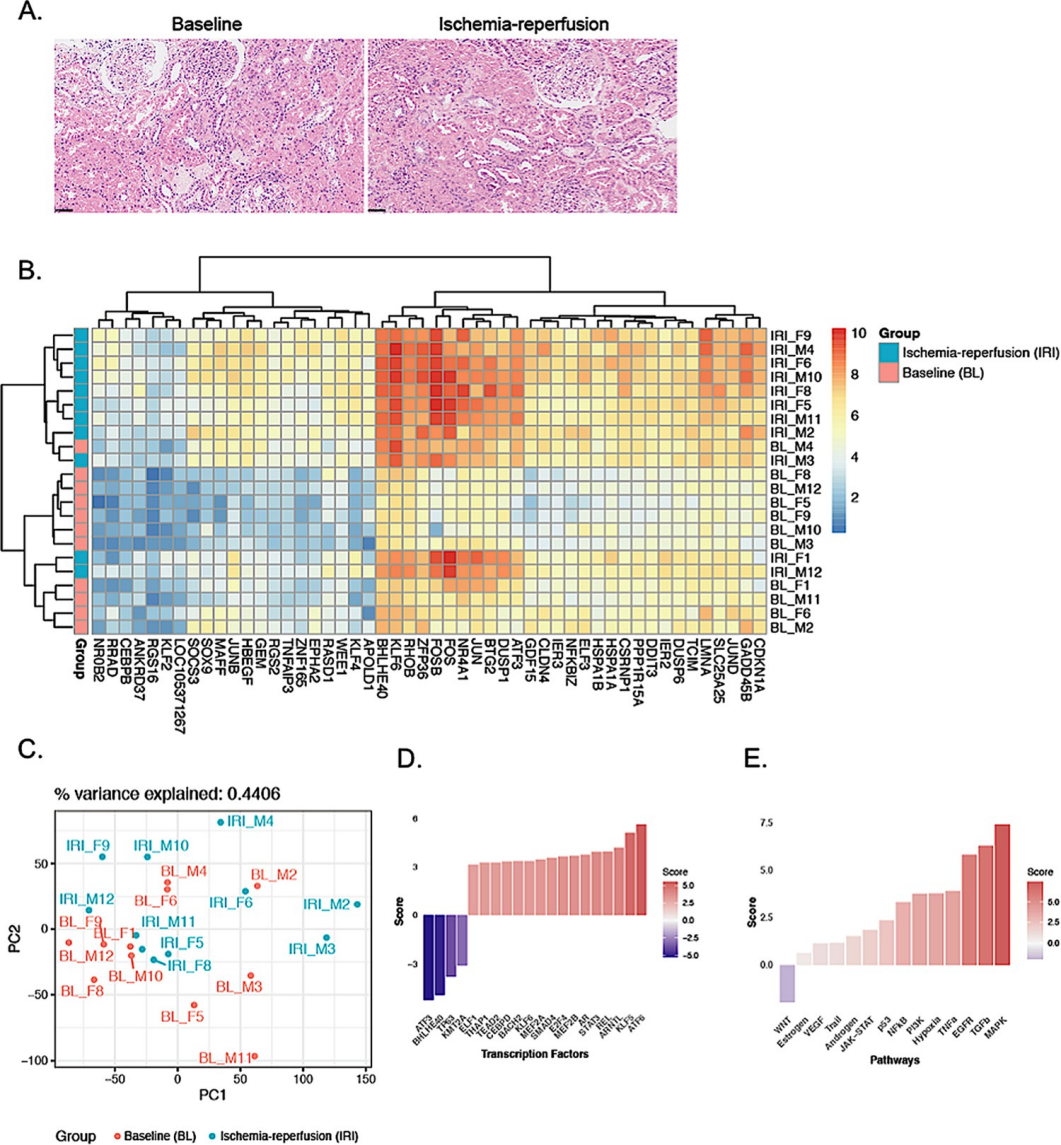
Immediate response to ischemia-reperfusion. (A) Histological features were similar between baseline and ischemia-reperfusion samples (HE staining, black bar = 50 μm). (B) Gene expression across samples for the top 50 significant differentially expressed genes (adjusted p-value < 0.05; |logFC| > 1.5). (C) Dimensionality reduction of the transcriptomic profiles across samples using the two first principal components revealed no clear separation between timepoints. (D) Transcription factor activities for the top 20 transcription factors that change the most in response to IRI. (E) Pathway activities in response to IRI.

The donor characteristics and graft outcomes in the IRI biopsy group are summarized in Table 1. A systematic histopathological examination of kidney biopsies in the IRI biopsy group revealed no major differences between pre- and post-ischemic samples in terms of interstitial fibrosis and tubular atrophy (IFTA), proximal tubular (PT) acute injury or vacuolization, cast formation or vascular changes, presented in S1 Table. However, the post-ischemic samples demonstrated slightly more severe ischemic changes than the pre-ischemic samples (Fig 2A).

**Table 1:** Demographics and clinical characteristics of the IRI biopsy group.

Variable	Donors (n = 11)	Recipients (n = 11)
Sex (F/M)	5/6	2/9
Age (year)	43,6 (11,3)	36,7 (16,8)
Weight (kg)	73,7 (13,4)	74,1 (25,0)
Height (cm)	174,8 (7,4)	168,0 (32,5)
BMI (kg/m^2^)	24,1 (3,6)	25,3 (4,4)
Pre-transplantation GFR	94,2 (9,4)	-
eGFR 12-month post-transplantation	-	72,8 (30,3)
Dialysis (PD/HD/No)	-	4/4/3
AB0 compatible (Yes/No)	-	7/4

Data are presented as mean (SD). GFR measured by iohexol clearance was available for donors. The estimated glomerular filtration rate (eGFR) was calculated for the recipients using the CKD-EPI (2021) equation. Abbreviations: *F* (Female), *M* (Male), *kg* (kilogram), *cm* (centimeter), *BMI* (Body Mass Index), *GFR* (Glomerular Filtration Rate), e*GFR* (estimated Glomerular Filtration Rate), *PD* (Peritoneal Dialysis), *HD* (Hemodialysis)

### Immediate transcriptomic response to ischemia-reperfusion in the IRI biopsy group

First, we characterized the immediate transcriptomic response to ischemia-reperfusion in living donor kidneys. After differential expression analysis, 244 genes were differentially expressed between pre- and post-IRI samples. A total of 239 genes were upregulated, and five genes were downregulated. With a more stringent filter for the False Discovery Rate, 69 genes remained upregulated, and no genes were downregulated. A list of all differentially expressed genes along with their statistics can be found in S2 Table. The 50 top differentially expressed genes are shown in the heat map in Fig 2B. In the unbiased principal component analysis (PCA) of the log-normalized counts (Fig 2C), there was no clear separation between pre- and post-IRI samples across the first two components (0.44% of variance explained).

### Transcription factor activity and pathway analysis in the IRI biopsy group

To gain a deeper understanding of the global transcriptomic effects of living-donor kidney transplantation, we performed an additional bioinformatics analysis. Inferred transcription factor activity revealed increased activity of several transcription factors in post-IRI samples, including ATF6, KLF5, AR and KLF6, but also decreased activity of ATF3 and BHLHE40 (Fig 2D). Pathway analysis indicated an increase in the activity of several pathways, including MAPK and EGFR in post-IRI samples compared to that in pre-IRI samples (Fig 2E). Only the Wnt pathway showed slightly reduced activity in the post-IRI samples.

### Consistency in the immediate response to IRI during kidney transplantation

To evaluate the robustness of the early transcriptomic response to ischemia-reperfusion, we performed a comparison with a previously published study [13] of paired kidney biopsies from living and deceased donors, which showed a significant overlap, where 50% (122/244) of the differentially expressed genes (DEGs) were differentially expressed in both datasets. A complete list of the genes in both studies is shown in S2 Table.

### Patient characteristics and histology in the RIPC biopsy group

**Fig 3:**
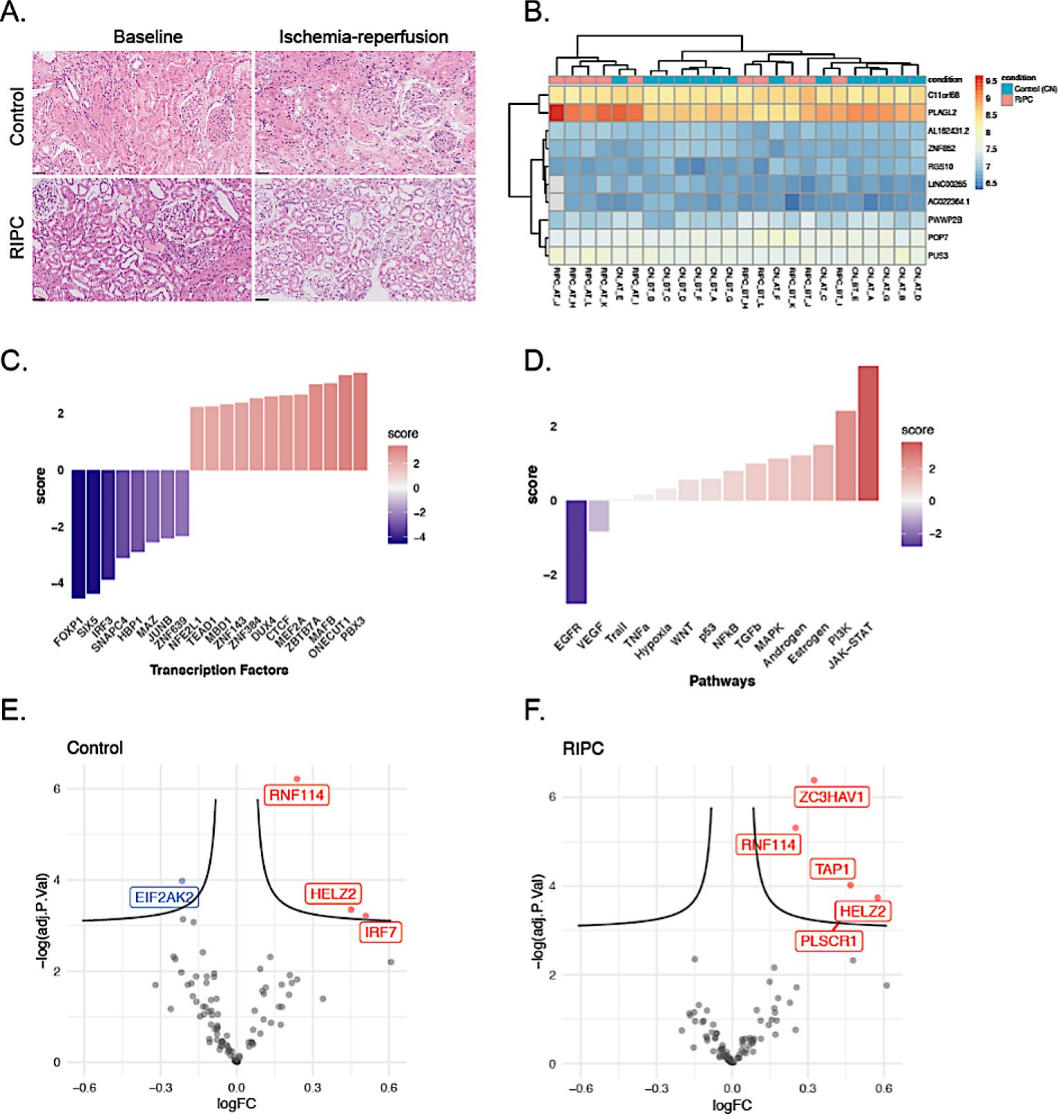
Modulation of the immediate response to IRI by RIPC. (A) Histological features were not affected by preconditioning with RIPC (HE staining, black bar = 50 μm). (B) Gene expression across samples for the top 10 significant differentially expressed genes (p-value < 0.05; |logFC| > 0.3). (C) Transcription factor activities for the top 20 transcription factors that changed the most in response to RIPC. (D) Pathway activities in response to RIPC. (E and F) Volcano plots of the target genes of the JAK-STAT pathway for IRI in Control and RIPC groups.

Next, we aimed to determine in an intervention study if a simple RIPC protocol can affect renal transcriptomic features of IRI. Baseline characteristics of donors and recipients and graft outcomes in the RIPC biopsy group are summarized in Table 2. A systematic histopathological examination of kidney biopsies in the RIPC cohort revealed no apparent effect of RIPC intervention on IFTA, PT acute injury or vacuolization, cast formation or vascular changes (Fig 3A and S3 Table).

**Table 2:** Demographics and clinical characteristics of the RIPC biopsy group.

Variable	Donors, no-pre (n = 7)	Donors, RIPC (N = 9)	Recipients, no-pre (n = 7)	Recipients, RIPC (n = 9)
Sex (F/M)	5/2	6/3	2/5	2/7
Age (year)	52,7 (11,8)	54,9 (12,0)	32,0 (8,3)	40,4 (15,9)
Weight (kg)	73,4 (12,3)	78,6 (12,6)	67,7 (12,3)	72,0 (8,4)
Height (cm)	169,3 (8,8)	170,3 (7,6)	172,4 (7,7)	176,3 (7,0)
BMI (kg/m^2^)	25,6 (4,3)	27,0 (3,0)	22,7 (2,9)	22,8 (1,9)
Pre-transplantation GFR	93,4 (7,1)	97,2 (7,6)	-	-
eGFR 12-month post-transplantation	-	-	54,1 (18,6)	69,7 (10,0) *
Dialysis (PD/HD/No)	-	-	1/1/5	4/1/4
AB0 compatible (Yes/No)	-	-	6/1	8/1

Demographics and clinical characteristics of donors and recipients in the RIPC biopsy group. Data are presented as mean (SD). GFR measured by iohexol clearance was available for donors. The estimated glomerular filtration rate (eGFR) was calculated for the recipients using the CKD-EPI (2021) equation. * p<0.05, between the no preconditioning and RIPC groups. Abbreviations: *no-pre* (no preconditioning).

### No modulation of the renal transcriptomic response to ischemia-reperfusion by RIPC

Overall, the transcriptomic response to IRI was very similar in donors preconditioned with RIPC and in donors without preconditioning, without any differences in differentially expressed genes. The top 20 genes ranked by p-value did not show a clear separation between the two groups (Fig 3C). After transcription factor activity inference, there was a trend towards higher activity in MAFB and PBX3 and less activity in FOXP1, SIX5, and IRF3 in donors subjected to RIPC as shown in Fig 3C. Pathway analysis revealed increased signaling in the JAK-STAT pathway and decreased signaling in the EGFR pathway following RIPC (Fig 3D–3F). In summary, we concluded that there was no apparent modulation of the transcriptomic response to IRI in living donor kidneys preconditioned with the standard RIPC protocol.

### Impact of RIPC on plasma metabolites in the RIPC blood group

**Fig 4:**
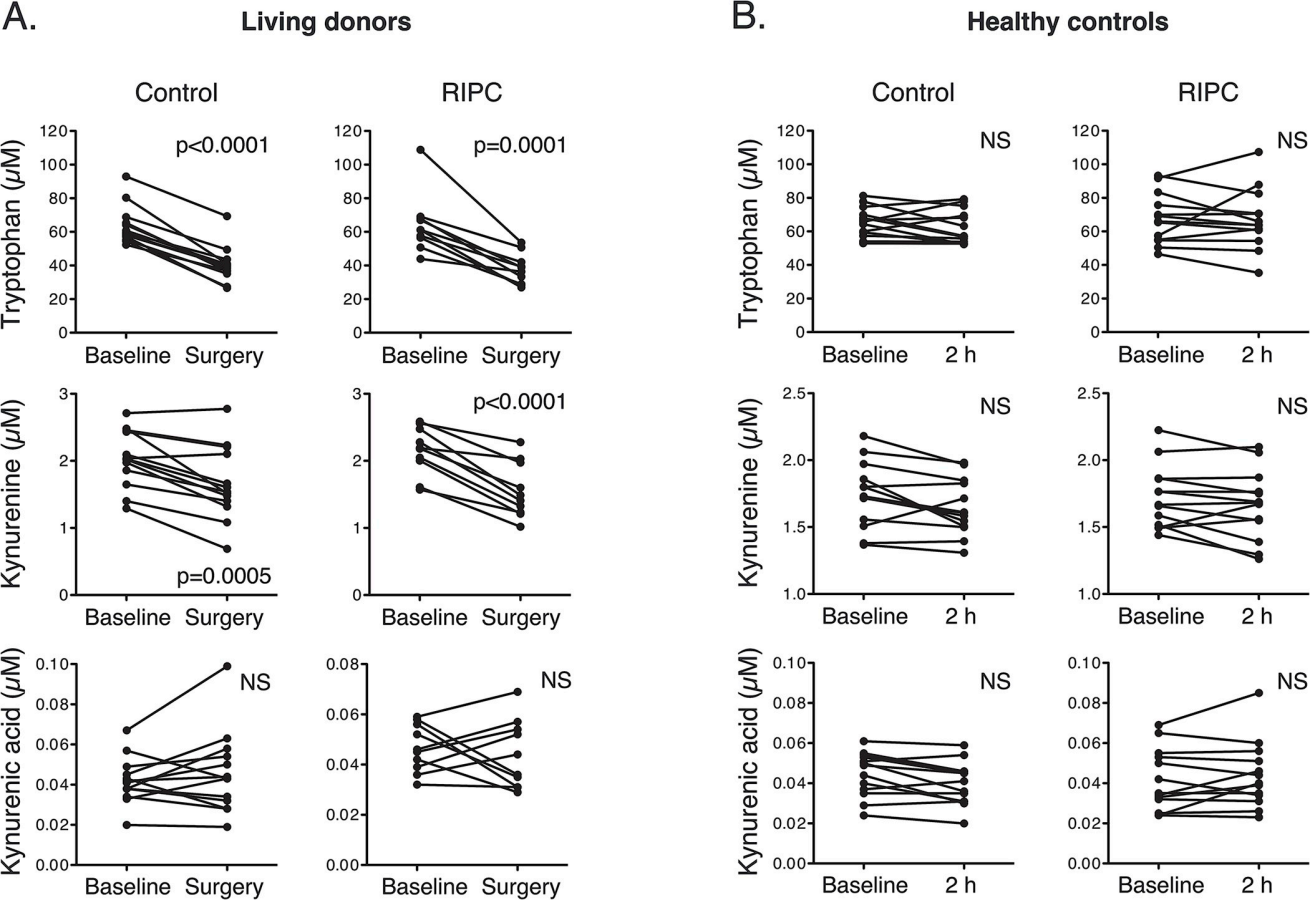
Plasma metabolites in response to RIPC. (A) Plasma metabolites in samples obtained from living donors before RIPC/no preconditioning and during surgery. (B) Plasma metabolites in samples obtained from healthy controls before RIPC/no preconditioning and 2 h after. All values are presented as μM.

In view of the negative results of the RIPC approach in modifying the transcriptomic response to IRI, the next aim was to assess whether the present RIPC protocol had meaningful biological effects on plasma metabolites in living donors treated with RIPC. KYNA has been suggested as an obligate humoral mediator of the protective effects of RIPC [14]. Therefore, we measured KYNA, tryptophan, kynurenine, and other metabolites in paired blood samples from 23 living donors. Blood samples were collected from both groups immediately prior to RIPC or no preconditioning. A second blood sampling from both groups was performed during transplantation surgery, approximately 2 h after RIPC or no preconditioning (Figs 1 and 4A, and Table 3). KYNA was unchanged between the two timepoints, both in donors subjected to RIPC and without preconditioning. Notably, concentrations of tryptophan, kynurenine, and several other metabolites decreased between the two timepoints, to a similar magnitude in both the RIPC and no preconditioning groups. To rule out the possibility that the lack of difference in KYNA after RIPC was due to the anesthesia or surgical procedures, as anesthesia has previously been reported to influence the effects of RIPC [15], we analyzed the same metabolites in healthy, awake controls subjected to either RIPC or no preconditioning, before and 2 h after the intervention (Fig 4B, Table 4, and S4 Table). RIPC did not cause an increase in blood concentration of KYNA or any of the other measured metabolites between the two timepoints in healthy awake subjects.

**Table 3:** Impact of RIPC/no preconditioning on plasma metabolites in living donors.

Metabolite	No-pre group, baseline	No-pre group, follow-up	*P-value*	RIPC group, baseline	RIPC group, follow-up	*P-value*
Phenylacetylglutamine	2.53	2.31	0.66	2.35	2.41	0.86
p-Cresol glucuronide	0.09	0.11	0.26	0.08	0.15	0.08
Phenylglucuronide	0.02	0.01	0.57	0.02	0.01	0.81
CMPF	6.53	5.36	0.00	8.43	6.83	0.00
Indoxyl sulfate	5.00	2.67	0.00	5.93	3.42	0.01
P-cresylsulphate	17.8	14.8	0.16	21.0	16.4	0.10
Hippuric acid	10.2	9.0	0.64	7.7	8.3	0.76
Phenylsulfate	4.76	3.11	0.18	4.19	2.57	0.10
Kynurenine	2.03	1.66	0.00	2.15	1.56	0.00
Tryptophan	63.7	40.4	0.00	64.4	37.9	0.00
Kynurenic acid	0.04	0.05	0.33	0.05	0.04	0.58
Tyrosine	73.0	56.3	0.00	77.3	56.6	0.03
Indole-3-acetic acid	1.89	1.32	0.01	1.97	1.32	0.00
Phenylalanine	72.5	62.6	0.01	73.4	58.4	0.00
TMAO	4.74	4.98	0.86	5.94	9.22	0.49
Phenylacetylglutamine	2.53	2.31	0.66	2.35	2.41	0.86

All values are presented as the mean values (μM). Abbreviations: *no-pre* (no preconditioning).

**Table 4:** Impact of RIPC/no preconditioning on plasma metabolites in healthy controls.

Metabolite	Control group, baseline	Control group, follow-up	*P-value*	RIPC group, baseline	RIPC group, follow-up	*P-value*
Phenylacetylglutamine	1.62	1.63	0.96	1.59	1.35	0.36
p-Cresol glucuronide	0.07	0.07	0.93	0.06	0.06	0.48
Phenylglucuronide	0.02	0.01	0.34	0.01	0.01	0.42
CMPF	4.18	4.26	0.50	5.39	5.30	0.59
Indoxyl sulfate	3.88	3.85	0.89	4.03	3.76	0.31
P-cresylsulphate	17.2	17.0	0.75	15.4	13.9	0.09
Hippuric acid	8.21	9.06	0.40	7.16	6.58	0.13
Phenylsulfate	3.85	3.57	0.09	2.56	2.30	0.10
Kynurenine	1.74	1.65	0.03	1.72	1.66	0.11
Tryptophan	65.6	62.7	0.25	67.6	67.1	0.89
Kynurenic acid	0.04	0.04	0.01	0.04	0.04	0.33
Tyrosine	62.8	59.3	0.22	73.2	76.1	0.49
Indole-3-acetic acid	1.75	1.76	0.94	1.75	1.55	0.09
Phenylalanine	79.0	73.0	0.04	83.6	85.2	0.50
TMAO	2.52	3.10	0.34	7.24	7.69	0.81
Phenylacetylglutamine	1.62	1.63	0.96	1.59	1.35	0.36

All values are presented as the mean values (μM).

## Discussion

In the first part of the present study, we demonstrated a clear pattern of IRI-induced changes in gene expression in biopsies from living kidney donors. In the second part of the study, we evaluated whether the response to IRI was significantly modulated by RIPC intervention and concluded that it was not. Finally, in the third part of the study, we report that RIPC intervention does not influence the blood concentration of KYNA and other metabolites in the kynurenine pathway. In summary, we validated living-donor kidney transplantation as a suitable model to study early changes related to ischemia-reperfusion and concluded that there is no evidence of a systemic impact of four episodes of 5 minute long RIPC on the upper arm, which is the standard method to induce RIPC in previously published clinical studies [4–7]. The observed lack of an effect suggests that a more robust protocol might be needed to achieve clinically meaningful results, and this consideration might help explain the inconsistent results of clinical trials evaluating RIPC.

Our data revealed that several transcription factors, including ATF6, KLF5 and -6, and AR, were activated in the kidneys after IRI. Notably, none of the hypoxia-inducible factors (including HIF1) showed significantly altered activity. Among the transcription factors with increased activity, ATF6 has been reported to reduce IRI in the heart and has been linked to endoplasmic reticulum and oxidative stress [16]. Similarly, KLF5 and KLF6 have been linked to ischemic injury in the brain and kidney, acting as targets for MiR-10b-3p and MiR-181d-5p, respectively [17, 18]. The androgen receptor (AR) has also been implicated in the response to cerebral ischemia and ischemic preconditioning and may account for some of the observed sex differences in response to IRI [19]. Interestingly, TP53 shows increased activity after IRI. TP53 is best known as a tumor suppressor gene but has also been evaluated as a therapeutic target in ischemic acute kidney injury, as it modulates the kidney response to IRI [20]. Two transcription factors, ATF3 and BHLHE40 exhibited decreased activity. ATF3 has previously been reported to be an endogenous inhibitor of myocardial IRI [21], and ATF3 overexpression reduces cardiac microvascular injury in rats [22]. Similarly, BHLHE40 was reported to be downregulated during cerebral ischemia, and its overexpression was shown to be protective [23]. Thus, we propose that the reactivation of ATF3 and BHLHE40 may be novel therapeutic targets to reduce IRI during kidney transplantation. RIPC intervention did not modulate the activity of any of the most upregulated or downregulated transcription factors after IRI.

Pathway analysis revealed the activation of several pathways, including MAPK signaling, upon IRI. The MAPK pathway was first implicated in the immediate response to renal ischemia-reperfusion 30 years ago [24] and was successfully targeted in both the injury and repair phases in experimental studies [25]. Additionally, various renoprotective treatments, such as RIPC and low-molecular-weight fucoidan, have been reported to suppress activation of the MAPK pathway in animal models of IRI [26, 27]. Therefore, the specific inhibition of MAPK signaling during kidney transplantation might pose another potential therapeutic avenue worth exploring.

Finally, mass spectrometry analysis revealed that circulating levels of KYNA, a proposed key humoral mediator of the protective effects of RIPC [14], were not altered in response to RIPC. Of note, plasma concentrations of seven of the 16 measured metabolites were lower in blood samples drawn during surgery than before surgery. The degree of change between the blood sampling timepoints was similar, regardless of ischemic preconditioning. In contrast, in awake healthy controls treated with RIPC, the plasma concentrations of these metabolites did not change between timepoints, suggesting that the observed changes in living kidney donors were due to anesthesia or the surgical intervention itself and not ischemic preconditioning.

We acknowledge that the present study has some limitations. This is a small pilot study that is not statistically powered to detect subtle changes induced by RIPC but is primarily intended to study whether there are any major modulations of the transcriptomic response to IRI induced by preconditioning. The study was not randomized or blinded, although we believe it is unlikely to affect the transcriptomic response to ischemia-reperfusion injury in any major way. Finally, the degree of ischemia is limited in living donors, and the results might have been different if kidneys from deceased donors exposed to more pronounced ischemia had been used.

In conclusion, we validated living-donor kidney transplantation as a robust and reproducible method for studying the immediate response to IRI in human kidneys. It is possible that new therapies to prevent or reduce IRI can be evaluated using this model. Importantly, we did not find that RIPC in living donors altered the early transcriptomic response to IRI or the serum levels of KYNA, as has previously been reported in animal studies. There may be several reasons why we did not observe a pronounced response to RIPC in our study. First, the method used to induce RIPC may be insufficient, as it only affects a relatively small group of muscles in one arm. A more robust induction of ischemic preconditioning, for example, by simultaneously preventing circulation to one arm and one leg or using a prolyl-hydroxylase inhibitor, might prove to be more effective. Second, the time point at which the kidney biopsies were taken (at 1 h after reperfusion in the recipient) might be suboptimal, and it is plausible that a more pronounced impact of RIPC could have been observed had the investigation been extended to a later time point.

RIPC holds promise for several indications, but due to inconsistent outcomes from clinical trials and the lack of understanding of the molecular mechanisms, it has yet to be implemented in clinical practice. Therefore, future studies should continue to elucidate the molecular mechanism underlying RIPC to enhance our understanding of how it should be used clinically to help protect patients from injury caused by ischemia-reperfusion, especially in situations with more severe ischemic insults, such as in organs donated from marginal donors.

## Supporting information

S1 Table Histology in the IRI biopsy group. X = no vessels. Gloms = Glomeruli.
(DOCX)

S2 Table Shared and specific genes in the present study and a previous study by Cippà PE et al. (DOCX)

S3 Table Histology in the RIPC biopsy group. X = no vessels. Gloms = Glomeruli.
(DOCX)

S4 Table Characteristics of the healthy awake participants in RIPC blood group. (DOCX)
