# Supplementary material for: Defining the molecular response to ischemia-reperfusion injury and remote ischemic preconditioning in human kidney transplantation
Source: PLoS One. 2024 Oct 29;19(10):e0311613. doi: 10.1371/journal.pone.0311613 (PMC11521294; doi:10.1371/journal.pone.0311613)
Supplement: S1 Table — X = no vessels. Gloms = Glomeruli. (DOCX) [file pone.0311613.s001.docx]

| **Biopsy** | **Comment** | **Gloms (n)** | **Gloms with sclerosis (n)** | **Inflammation (1-3)** | **IF** **(1-3)** | **TA** **(1-3)** | **Hyalinos** **(1-3)** | **Intimal fibrosis (1-3)** | | **Tubular flattening (1-3)** | | **Tubular vacuolization (1-3)** | | **Tubular cell debris (1-3)** | | **Degree of ischemia** | |  |
| --- | --- | --- | --- | --- | --- | --- | --- | --- | --- | --- | --- | --- | --- | --- | --- | --- | --- | --- |
| 1 Pre-ischemia |  | 5 | 0 | 0 | 0 | 0 | 0 | | X | | 1 | | 1 | | 1 | | Mild | |
| 1 Post-ischemia |  | 16 | 0 | 0 | 0 | 0 | 0 | | X | | 1 | | 0 | | 1 | | Mild | |
| 2 Pre-ischemia |  | 5 | 0 | 0 | 0 | 0 | 0 | | X | | 1 | | 1 | | 2 | | Mild | |
| 2 Post-ischemia | Missing |  |  |  |  |  |  | |  | |  | |  | |  | |  | |
| 3 Pre-ischemia |  | 16 | 0 | 0 | 0 | 0 | 1 | | 0 | | 1 | | 0 to 1 | | 2 | | Mild | |
| 3 Post-ischemia |  | 3 | 0 | 0 |  | 0 | 0 | | X | | 1 | | 2 | | 2 | | Moderate | |
| 4 Pre-ischemia |  | 6 | 1 | 0 | 1 | 1 | 0 | | 0 | | 0 | | 0 | | 1 | | Mild | |
| 4 Post-ischemia |  | 15 | 0 | 0 | 1 | 1 | 0 | | X | | 0 | | 0 | | 1 | | Mild | |
| 5 Pre-ischemia |  | 5 | 0 | 0 | 1 | 0 | 0 | | X | | 1 | | 0 | | 2 | | Moderate | |
| 5 Post-ischemia |  | 10 | 0 | 0 | 0 | 0 | 0 | | 0 | | 1 | | 0 | | 1 | | Mild | |
| 6 Pre-ischemia |  | 9 | 0 | 0 | 1 | 0 | 0 | | X | | 1 | | 0 | | 2 | | Mild | |
| 6 Post-ischemia |  | 12 | 1 | 0 | 1 | 0 | 0 | | 0 | | 2 | | 1 | | 2 | | Moderate | |
| 7 Pre-ischemia |  | 13 | 0 | 0 | 0 | 0 | 0 | | 0 | | 2 | | 0 | | 1 | | Mild | |
| 7 Post-ischemia |  | 14 | 0 | 0 | 0 | 0 | 0 | | 0 | | 2 | | 0 | | 2 | | Moderate | |
| 8 Pre-ischemia |  | 17 | 2 | 0 | 1 | 0 | 0 | | 0 | | 1 | | 0 | | 0 | | Mild | |
| 8 Post-ischemia |  | 12 | 2 | 0 | 1 | 1 | 2 | | 1 | | 1 | | 0 | | 1 | | Mild | |
| 9 Pre-ischemia |  | 8 | 5 | 0 | 1 | 1 | 0 | | 0 | | 1 | | 0 | | 1 | | Mild | |
| 9 Post-ischemia |  | 9 | 0 | 0 | 1 | 0 | 0 | | 0 | | 2 | | 0 | | 2 | | Moderate | |
| 10 Pre-ischemia |  | 6 | 0 | 0 | 0 | 0 | 0 | | 2 | | 1 | | 2 | | 2 | | Mild | |
| 10 Post-ischemia |  | 24 | 0 | 0 | 0 | 0 | 0 | | 0 | | 2 | | 1 | | 2 | | Moderate | |
| 11 Pre-ischemia |  | 19 | 0 | 0 | 1 | 1 | 0 | | 1 | | 2 | | 0 | | 2 | | Moderate | |
| 11 Post-ischemia | Missing |  |  |  |  |  |  | |  | |  | |  | |  | |  | |

# Supporting information

**S1 Table.** **Histology in the IRI biopsy group.** X=no vessels. Gloms=Glomeruli.
