# Supplementary material for: Defining the molecular response to ischemia-reperfusion injury and remote ischemic preconditioning in human kidney transplantation
Source: PLoS One. 2024 Oct 29;19(10):e0311613. doi: 10.1371/journal.pone.0311613 (PMC11521294; doi:10.1371/journal.pone.0311613)
Supplement: S2 Table — (DOCX) [file pone.0311613.s002.docx]

# Supporting information

**S2 Table.** **Shared and specific genes in the present study and a previous study by Cippà PE et al.**

| **Specific for Nordström J et al** | **Specific for Cippà PE et al.** | **Shared** |
| --- | --- | --- |
| HSPA1B | CCNL1 | RHOB |
| HSPA1A | DNAJB1 | KLF4 |
| LOC105371267 | TRIB1 | NR0B2 |
| ZNF165 | PMAIP1 | APOLD1 |
| TCIM | RIPK4 | KLF2 |
| IER3 | MCL1 | PPP1R15A |
| CXCR4 | DNAJB4 | DUSP1 |
| FOXQ1 | NFIL3 | CEBPB |
| TNFRSF12A | PIM1 | FOSB |
|  | CYR61 | BHLHE40 |
|  | EGR3 | SLC25A25 |
|  | IFRD1 | DUSP6 |
|  | CXCL2 | ZFP36 |
|  | SERPINE1 | NFKBIZ |
|  | SLC20A1 | RASD1 |
|  | MAP3K8 | KLF6 |
|  | RND3 | RRAD |
|  | JMJD6 | DDIT3 |
|  | TGIF1 | GADD45B |
|  | ZFP36L1 | JUNB |
|  | MXD1 | SOCS3 |
|  | DNAJA1 | RGS16 |
|  | TIPARP | ANKRD37 |
|  | HSP90AA1 | FOS |
|  | OSR2 | ATF3 |
|  | DNAJA4 | HBEGF |
|  | HSP90AB1 | CSRNP1 |
|  | RND1 | IER2 |
|  | CEBPD | GDF15 |
|  | DUSP10 | GEM |
|  | PLK2 | CLDN4 |
|  | NR4A3 | JUN |
|  | IER5 | CDKN1A |
|  | HSPA8 | JUND |
|  | AREG | EPHA2 |
|  | DUSP2 | RGS2 |
|  | HSPH1 | NR4A1 |
|  | IL6 | ELF3 |
|  | SELE | BTG2 |
|  | BCL6 | SOX9 |
|  | BAG3 | WEE1 |
|  | PER1 | LMNA |
|  | VPS37B | MAFF |
|  | GADD45G | TNFAIP3 |
|  | NR1D1 | MYC |
|  | TSPYL2 | PLK3 |
|  | SERPINH1 | ZC3H12A |
|  | EDN1 | SERTAD1 |
|  | ZFAND2A | SLC2A3 |
|  | ITPKC | EGR1 |
|  | BTBD19 | LDLR |
|  | CCL2 | ICAM1 |
|  | ADAMTS1 | DUSP5 |
|  | PER2 | BCL3 |
|  | ELMSAN1 | RASGEF1B |
|  | ARID5A | IRF1 |
|  | PTGS2 | LIF |
|  | CXCL1 | EMP1 |
|  | GATA6 | NR4A2 |
|  | IL1B | EGR2 |
|  | PPP1R15B |  |
|  | C8orf4 |  |
|  | CLK1 |  |
|  | AC007906.2 |  |
|  | TSC22D1 |  |
|  | KLF10 |  |
|  | ARL5B |  |
|  | NFKBIA |  |
|  | NCOA7 |  |
|  | B3GNT5 |  |
|  | HES1 |  |
|  | MYADM |  |
|  | PNRC1 |  |
|  | RGS1 |  |
|  | C10orf10 |  |
|  | TOB1 |  |
|  | NUAK2 |  |
|  | LETM2 |  |
|  | C11orf96 |  |
|  | ODF3L1 |  |
|  | CXCL3 |  |
|  | NEDD9 |  |
|  | CD83 |  |
|  | CLDN14 |  |
|  | ARL4A |  |
|  | CKS2 |  |
|  | SNAI1 |  |
|  | CHORDC1 |  |
|  | CD69 |  |
|  | TSC22D3 |  |
|  | FILIP1L |  |
|  | CITED2 |  |
|  | TNFRSF10D |  |
|  | LINC00675 |  |
|  | MT1A |  |
|  | IGFBP1 |  |
|  | ADAMTS4 |  |
|  | ANXA1 |  |
|  | HIST1H2BC |  |
|  | GPR132 |  |
|  | HBB |  |
|  | ADM |  |
|  | NOCT |  |
|  | BIRC3 |  |
|  | ZNF878 |  |
|  | NEURL3 |  |
|  | ERRFI1 |  |
|  | MB21D1 |  |
|  | PDK4 |  |
|  | OASL |  |
|  | LEAP2 |  |
|  | GPR183 |  |
|  | PTX3 |  |
|  | NLRP3 |  |
|  | ADRB2 |  |
|  | HBA1 |  |
|  | CCL8 |  |
|  | HBA2 |  |
|  | HAS2 |  |
|  | FGA |  |
|  | LRG1 |  |
|  | EDN2 |  |
|  | SERPINA3 |  |
|  | TAGAP |  |
|  | MMP19 |  |
|  | RGS17 |  |
|  | S100A8 |  |
|  | CCL20 |  |
|  | CGA |  |
|  | ANGPTL4 |  |
|  | CXCR2 |  |
|  | FCN1 |  |
|  | CCR2 |  |
|  | PLAC8 |  |
|  | RSPO3 |  |
|  | FGG |  |
|  | MS4A1 |  |
|  | IGKV1-5 |  |
|  | IGKV3-11 |  |
