# Supplementary material for: Defining the molecular response to ischemia-reperfusion injury and remote ischemic preconditioning in human kidney transplantation
Source: PLoS One. 2024 Oct 29;19(10):e0311613. doi: 10.1371/journal.pone.0311613 (PMC11521294; doi:10.1371/journal.pone.0311613)
Supplement: S3 Table — X = no vessels. Gloms = Glomeruli. (DOCX) [file pone.0311613.s003.docx]

# Supporting information

**S3 Table. Histology in the RIPC biopsy group.** X=no vessels. Gloms=Glomeruli.

| **Biopsy** | **Comment** | **Gloms (n)** | **Gloms with sclerosis (n)** | **Inflammation (1-3)** | **IF** **(1-3)** | **TA** **(1-3)** | **Hyalinos** **(1-3)** | **Intimal fibrosis (1-3)** | **Tubular flattening (1-3)** | | **Tubular vacuolization (1-3)** | | **Tubular cell debris (1-3)** | **Degree of ischemia** |
| --- | --- | --- | --- | --- | --- | --- | --- | --- | --- | --- | --- | --- | --- | --- |
| 1 Pre-ischemia | Control group | 21 | 0 | 0 | 0 | 0 | 0 | 0 | 0 | 1 | |  | 0 | None |
| 1 Post-ischemia | Control group | 10 | 0 | 0 | 0 | 0 | 0 | 0 | 1 | 0 | |  | 2 | Mild |
| 2 Pre-ischemia | Control group | 20 | 0 | 0 | 0 | 0 | 0 | 0 | 1 | 0 | |  | 2 | Mild |
| 2 Post-ischemia | Control group | 13 | 2 | 0 | 0 | 1 | 0 | 0 | 3 | 1 | |  | 3 | Moderate |
| 3 Pre-ischemia | Control group | 10 | 1 | 0 | 0 | 0 | 0 | 0 | 1 | 0 | |  | 1 | Mild |
| 3 Post-ischemia | Control group | 4 | 0 | 0 | 0 | 0 | 0 | 0 | 2 | 3 | |  | 2 | Severe |
| 4 Pre-ischemia | Control group | 6 | 0 | 0 | 0 | 0 | 0 | 0 | 1 | 0 | |  | 2 | Moderate |
| 4 Post-ischemia | Control group | 7 | 0 | 0 | 0 | 0 | 0 | 1 | 1 | 0 | |  | 1 | Mild |
| 5 Pre-ischemia | Control group | 9 | 0 | 0 | 0 | 0 | 0 | 0 | 1 | 0 | |  | 1 | Mild |
| 5 Post-ischemia | Control group | 21 | 0 | 0 | 0 | 0 | 0 | 0 | 1 | 1 | |  | 2 | Severe |
| 6 Pre-ischemia | Control group | 18 | 0 | 0 | 0 | 0 | 0 | 0 | 0 | 0 | |  | 0 | None |
| 6 Post-ischemia | Control group | 18 | 0 | 0 | 0 | 0 | 0 | 1 | 0 | 1 | |  | 1 | None |
| 7 Pre-ischemia | Control group | 12 | 1 | 0 | 0 | 0 | 1 | 1 | 2 | 0 | |  | 2 | Moderate |
| 7 Post-ischemia | Control group | 9 | 1 | 0 | 1 | 0 | 1 | X | 1 | 0 | |  | 2 to 3 | Severe |
| 1 Pre-ischemia | RIPC group | 18 | 0 | 0 | 1 | 1 | 1 | 0 | 2 | 0 | |  | 2 | Moderate |
| 1 Post-ischemia | RIPC group | 6 | 0 | 0 | 1 | 0 | 1 | X | 2 | 2 | |  | 2 to 3 | Severe |
| 2 Pre-ischemia | RIPC group | 10 | 3 | 0 | 1 | 0 | 0 | 1 | 1 | 1 | |  | 2 | Moderate |
| 2 Post-ischemia | RIPC group | 14 | 0 | 0 | 0 | 0 | 1 | 0 | 1 | 1 | |  | 2 | Severe |
| 3 Pre-ischemia | RIPC group | 9 | 1 | 0 | 0 | 0 | 0 | X | 0 | 0 | |  | 0 | None |
| 3 Post-ischemia | RIPC group | 16 | 0 | 0 | 0 | 0 | 0 | 0 | 0 | 0 | |  | 0 to 1 | Mild |
| 4 Pre-ischemia | RIPC group | 6 | 0 | 0 | 0 | 0 | 0 | X | 1 | 0 | |  | 1 | Mild |
| 4 Post-ischemia | RIPC group | 5 | 0 | 0 | 0 | 0 | 0 | 1 | 1 | 1 | |  | 2 | Moderate |
| 5 Pre-ischemia | RIPC group | 14 | 0 | 0 | 0 | 0 | 1 | 0 | 1 | 0 | |  | 2 | Mild |
| 5 Post-ischemia | RIPC group | 9 | 1 | 0 | 0 | 0 | 0 | 0 | 1 | 0 | |  | 2 | Moderate |
| 6 Pre-ischemia | RIPC group | 20 | 1 | 0 | 0 | 0 | 0 | 0 | 2 | 0 | |  | 1 | Mild |
| 6 Post-ischemia | RIPC group | 25 | 0 | 0 | 1 | 0 | 0 | 0 | 2 to 3 | 0 | |  | 2 | Moderate |
| 7 Pre-ischemia | RIPC group | 24 | 0 | 0 | 1 | 0 | 0 | 0 | 2 | 0 | |  | 2 | Moderate |
| 7 Post-ischemia | RIPC group | 6 | 0 | 0 | 0 | 0 | 0 | 0 | 2 | 0 | |  | 2 | Moderate |
| 8 Pre-ischemia | Missing |  |  |  |  |  |  |  |  |  | |  |  |  |
| 8 Post-ischemia | RIPC group | 12 | 0 | 0 | 0 | 0 | 1 | 1 | 3 | 0 | |  | 2 | Moderate |
| 9 Pre-ischemia | RIPC group | 14 | 1 | 0 | 0 | 0 | 0 | 0 | 0 to 1 | 0 | |  | 1 | Mild |
| 9 Post-ischemia | RIPC group | 9 | 0 | 0 | 0 | 0 | 0 | 1 | 2 | 0 | |  | 2 | Severe |
