# Supplementary material for: Defining the molecular response to ischemia-reperfusion injury and remote ischemic preconditioning in human kidney transplantation
Source: PLoS One. 2024 Oct 29;19(10):e0311613. doi: 10.1371/journal.pone.0311613 (PMC11521294; doi:10.1371/journal.pone.0311613)
Supplement: S4 Table — (DOCX) [file pone.0311613.s004.docx]

# Supporting information

**S4 Table.** **Characteristics of the healthy awake participants in RIPC blood group.**

|  | RIPC (n=13) | Control (n=13) |
| --- | --- | --- |
| Female (n) | 8 | 9 |
| Male (n) | 5 | 4 |
| Age (y, average) | 34 | 38 |
